# Identification of crosstalk genes and diagnostic biomarkers in systemic sclerosis associated sarcopenia through integrative analysis and machine learning

**DOI:** 10.3389/fimmu.2025.1642806

**Published:** 2025-09-05

**Authors:** Yanfang Wu, Yunfeng Dai, Fei Gao, Haiping Xie, Shuyao Pan, Juanjuan He, Jianwen Liu, He Lin, Zhihan Chen, Junping Wen

**Affiliations:** ^1^ Shengli Clinical Medical College of Fujian Medical University, Fuzhou, China; ^2^ Department of Rheumatology, Fuzhou University Affiliated Provincial Hospital, Fuzhou, China; ^3^ Department of Endocrinology, Fuzhou University Affiliated Provincial Hospital, Fuzhou, China

**Keywords:** systemic sclerosis, sarcopenia, biomarker, NOX4, NEK6, machine learning, immune infiltration

## Abstract

**Background:**

Sarcopenia associated with systemic sclerosis (SSc) significantly compromises patient prognosis and quality of life. However, reliable diagnostic biomarkers remain lacking. This study aimed to identify molecular markers for early detection using integrative computational approaches.

**Methods:**

An integrated analysis based on the Gene Expression Omnibus (GEO) database was performed. Crosstalk genes (CGs) were identified using least absolute shrinkage and selection operator (LASSO) regularization, ensemble decision trees, and support vector machine-based feature selection. Machine learning algorithms were employed to construct a predictive scoring model and to assess the diagnostic value of key biomarkers. Hub mRNAs were validated using quantitative polymerase chain reaction (qPCR). Immune cell infiltration profiles and functional correlations were also examined.

**Results:**

Five key CGs—NOX4, STC2, NEK6, IGSF10, and EMX2—were identified as molecular links between SSc and sarcopenia. A predictive model incorporating NOX4 and NEK6 was developed, and a diagnostic threshold was established. PCR validation confirmed the differential expression of NOX4 and NEK6 in both SSc and SSc-associated sarcopenia, demonstrating high predictive accuracy. Furthermore, the combined NOX4-NEK6 model exhibited a superior area under the curve (AUC) compared to either gene alone. Immune infiltration analysis revealed significant correlations between CGs and multiple immune cell populations.

**Conclusion:**

This study proposes NOX4 and NEK6 as novel biomarkers, offering a non-invasive strategy for the early detection of SSc-associated sarcopenia. This study also reveals a shared immune-dysregulation node linking SSc and sarcopenia, positions these crosstalk genes as multi-disease prevention targets, and paves the way for personalized immunotherapy and rapid bench-to-bedside translation.

## Introduction

Systemic sclerosis (SSc) is a rare autoimmune disorder characterized by fibrosis of the skin and internal organs, vasculopathy, and immune dysregulation (1). In recent years, sarcopenia—defined by the progressive loss of skeletal muscle mass and function—has garnered increasing attention (2). The prevalence of sarcopenia among patients with SSc is significantly higher than in the general population, with reported rates ranging from 20.7% to 54.8% (3–7), and 44% in our previous study. In SSc, high levels of pro-inflammatory cytokines like IL - 6 and TNF-α contribute to muscle loss. Excess ROS leads to apoptosis and neuromuscular junction failure in myopenia, while also promoting sarcopenic anabolic resistance by disrupting key protein signaling. Importantly, SSc-associated sarcopenia not only severely impairs quality of life (8), but is also closely associated with rapid disease progression and increased in-hospital mortality (9), highlighting the urgent need for mechanistic insights and early intervention strategies.

Two major limitations currently hinder the clinical diagnosis of sarcopenia. Dual-energy X-ray absorptiometry (DXA), considered the gold standard for assessing muscle mass, is restricted by the need for specialized equipment and technical expertise (10). While bioelectrical impedance analysis (BIA) offers greater convenience, its accuracy is affected by variables such as hydration status (11). Functional evaluations, including isometric grip strength and the five-times sit-to-stand test, are valuable but also require standardized procedures and tools (12, 13). These limitations have spurred the search for novel biomarkers and intelligent assessment systems. In recent years, there has been a growing interest in biomarkers that may have diagnostic value for sarcopenia. The indicators that have been found include those linked to hormones, metabolism, inflammation, genetics, amino acids, and more (14). With a modest level of diagnostic accuracy, the creatinine to cystatin C ratio (Cr/CysC) has become the most commonly used diagnostic biomarker among them. Nevertheless, there is variation in the diagnostic performance of these common biomarkers for sarcopenia depending on the diagnostic criteria. Furthermore, people with coronary heart disease, chronic kidney disease, type 2 diabetes, and the elderly make up the majority of the populations under study (15). Research on biomarkers for sarcopenia linked to systemic sclerosis is currently lacking.

Current therapeutic strategies predominantly involve exercise interventions and nutritional supplementation, with resistance training combined with protein or β-hydroxy-β-methylbutyrate (HMB) supplementation recognized as the gold standard (16). However, clinical evidence suggests that nutritional support alone yields limited improvements in muscle function and must be paired with structured exercise programs to achieve therapeutic efficacy (17). Innovative non-pharmacological interventions—such as gut microbiota modulators (probiotics/prebiotics), whole-body vibration therapy, blood flow restriction training, and neuromuscular electrical stimulation—offer alternative options for patients with exercise limitations (18). Nonetheless, these approaches often suffer from delayed efficacy and poor patient adherence, underscoring the need for targeted therapies based on underlying pathophysiological mechanisms.

Advancements in histological techniques have enabled gene expression profiling to become a powerful tool for elucidating the mechanisms of SSc-associated sarcopenia. In this study, we aimed to identify crosstalk genes, along with differentially expressed genes (DEGs) that were dysregulated in both SSc and sarcopenia and to construct a robust genetic prediction model. We further validated the identified biomarkers and risk model in our own cohort. These findings offer promising avenues for early diagnosis and provide a foundation for investigating the mechanistic interactions underlying SSc and sarcopenia.

## Methods

### Data sources

Microarray datasets were obtained from the Gene Expression Omnibus (GEO) database (http://www.ncbi.nlm.nih.gov/geo/) (19), which hosts a wide range of high-throughput sequencing and gene expression data. The terms “systemic sclerosis” and “sarcopenia” were used as search keywords to identify relevant gene expression datasets, and non-human samples were excluded.

The GSE181549 dataset (20), based on the GPL13497 platform, includes 113 skin samples from SSc patients and 44 from healthy controls. The GSE167186 dataset (21), generated using the GPL20301 platform, comprises 72 skin samples, including 24 from patients with sarcopenia and 48 from healthy controls. All patients with SSc met the diagnostic criteria established by the American College of Rheumatology (ACR) (22), and sarcopenia diagnoses were based on the guidelines set by the European Working Group on Sarcopenia in Older People (EWGSOP) (23).

**Figure 1:**
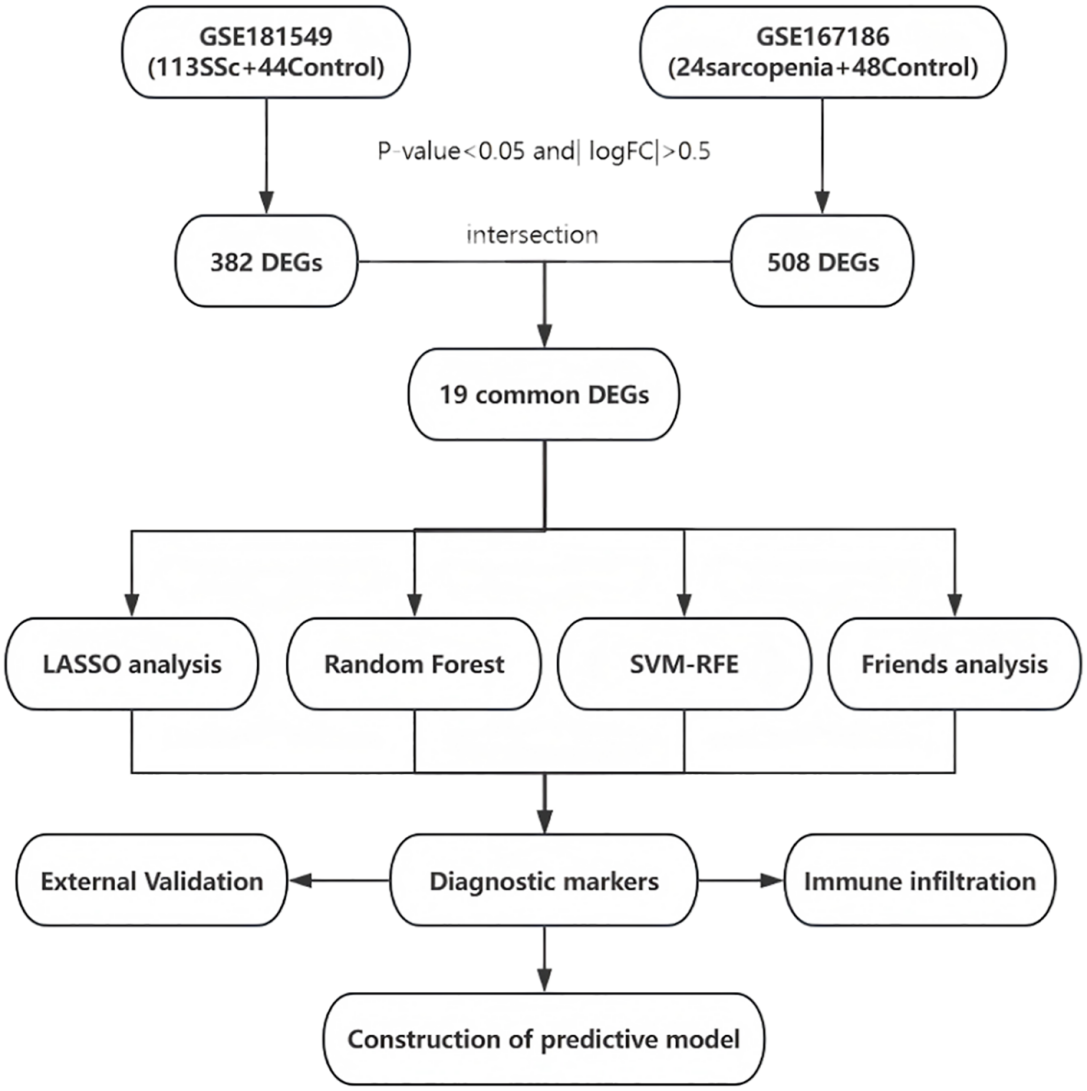
Entire working processes of the study. SSc, Systemic sclerosis; DEGs, differently expressed genes; LASSO, Least Absolute Shrinkage and Selection Operator; SVM-RFE, Support Vector Machine - Recursive Feature Elimination.

As these datasets are publicly available and freely accessible, no ethics committee approval was required. The complete workflow is illustrated in **Figure 1**.

### Identification of differentially expressed genes

To ensure that the same gene was compared in both datasets, we first converted Agilent probe IDs (GSE181549) and Illumina Ensembl IDs (GSE167186) to official HGNC gene symbols using the most recent platform annotation files. Only genes unambiguously mapped to identical symbols in both datasets were retained. DEGs were identified separately from the GSE181549 and GSE167186 datasets using the “limma” R package. Benjamini and Hochberg’s approach was used to adjust P-values for controlling the false discovery rate (FDR). Genes with a adjusted P-value < 0.05 and an absolute log fold change (|logFC|) > 0.5 were considered statistically significant. The results were visualized through gene clustering heatmaps and volcano plots. A comparative analysis of DEGs from the two datasets was performed, and overlapping genes potentially involved in the pathogenesis of both SSc and sarcopenia were identified using a Venn diagram.

### Machine learning algorithms

Further screening of the shared genes between SSc and sarcopenia was conducted using three widely accepted machine learning algorithms: Least Absolute Shrinkage and Selection Operator (LASSO), Random Forest (RF), and Support Vector Machine - Recursive Feature Elimination (SVM-RFE).

Initially, the 19 previously identified common genes were input into the LASSO algorithm within the SSc dataset. A regression model was constructed using the “glmnet” R package with 10-fold cross-validation and a seed value of 100. The “family” parameter was set to “binomial,” and the optimal value of the regularization parameter was determined using “lambda.1min.” The log(λ) profiles of the LASSO coefficients for the 19 features were plotted, followed by the generation of the binomial deviance curve and the log(λ) curve. The optimal value for 1 standard error (1-SE) from the minimum criterion was subsequently determined.

Next, the Random Forest algorithm was implemented using the R package randomForest (v4.7-1.1). The model was trained on the SSc cohort with the following parameters: ntree = 500 (number of trees grown), mtry = floor(sqrt(p)) = 4 (number of variables randomly sampled as candidates at each split, where p=19 is the total number of input genes), nodesize = 1 (minimum size of terminal nodes) and the Gini impurity criterion. To ensure reproducibility, a random seed was set to 100. Variable importance was assessed with the Gini impurity decrease (MeanDecreaseGini), and the top 10 predictors were visualized.

Finally, SVM-RFE recursively eliminated the least important features based on their SVM weight coefficients while conducting cross-validation to evaluate model accuracy and error rates (24). This algorithm was performed using the e1071 package (v1.7-14) with default parameters: radial basis function kernel, cost = 1, and gamma = 1/19. The algorithm recursively eliminated features with the smallest absolute weight coefficients. Ten-fold cross-validation monitored prediction accuracy across progressively smaller feature subsets (from 19 to 1 feature), with the subset achieving minimum cross-validation error selected as optimal.

Genes identified by the intersection of all three machine learning algorithms, visualized through a Venn diagram, were selected as the diagnostic gene targets for SSc-associated sarcopenia.

Additionally, a similarity analysis of the common DEGs was performed using the “GOSemSim” R package to compare these genes with reference genomes. The geometric mean of Gene Ontology (GO) semantic similarity scores—encompassing biological process, molecular function, and cellular component—was calculated. The results were illustrated using a cloud and rain plot generated by the “ggplot2” R package.

### Construction and validation of the SSc-associated sarcopenia risk score

The expression levels of candidate diagnostic genes were evaluated separately in the SSc and sarcopenia cohorts. The diagnostic utility of these biomarkers was assessed using receiver operating characteristic (ROC) curve analysis, and the area under the curve (AUC) was calculated.

Multivariate logistic regression analysis was conducted to construct a risk score for SSc-associated sarcopenia based on the shared diagnostic genes. For each sample, the risk score was computed using the gene expression levels and the corresponding logistic regression coefficients. The formula for the risk score was defined as follows:


$$\text{Risk score }=\sum_{i}coefficient\_i\times Expression\_i$$

where Coefficient_i represents the regression coefficient for gene i, and Expression_i denotes the expression level of gene i in each patient.

A nomogram was constructed using the “rms” R package to enhance the clinical applicability of the risk model. The performance of the model was evaluated through ROC curve analysis and AUC values. Model calibration and predictive consistency were assessed using calibration curves and concordance indexes (C-index).

To ensure the internal validation of model performance and guard against the risk of overfitting, this study adopted a 5-fold cross-validation strategy in the systemic sclerosis dataset. We repeated the entire 5-fold cross-validation process 200 times. During each iteration of cross-validation, we meticulously calculated key performance metrics, including the Discriminant Index (Dxy), AUC value, Coefficient of Determination (R^2^), and Brier score. Finally, we aggregated the mean values of all performance metrics to obtain a comprehensive assessment of the model’s performance.

### Verification of Hub mRNAs using quantitative PCR

Peripheral blood samples were collected from 32 SSc patients and 20 healthy individuals at Fujian Provincial Hospital. All patients were diagnosed according to the ACR classification criteria for SSc (22). The study protocol was approved by the Ethics Committee of Fujian Provincial Hospital. All procedures complied with the Declaration of Helsinki, and written informed consent was obtained from all participants.

qPCR was performed to validate the expression levels of the hub mRNAs, NOX4 and NEK6. Total RNA was extracted using TRI REAGENT BD (MRCGENE) according to the manufacturer’s instructions. RNA was then reverse transcribed into complementary DNA (cDNA) using the GeneAmp PCR System 9700 (Applied Biosystems). qPCR was conducted on a QuantStudio™ 5 Real-Time PCR System (Applied Biosystems), using GAPDH as the internal control. The relative expression levels of target genes were calculated using the 2^−ΔΔCt^ method. Primer sequences for the hub genes are listed in **Table 1**.

**Table 1:** Primer sequences used for real-time quantitative PCR.

m-RNA	Primers
GAPDH	F:5’GGGAAACTGTGGCGTGAT3’ R:5’GAGTGGGTGTCGCTGTTGA3’
NOX4	F:5’ CAGTCAAACAGATGGGATACAGA 3’ R:5’ GTCCACAACAGAAAACACCAAC3’
NEK6	F:5’ ACCCACAGAGGCATCCCAAC 3’ R:5’ CCTTGGCGTCCATCATCTCA 3’

Using PCR data obtained from our center as the validation set, we assessed the diagnostic performance of NOX4, NEK6, and a dual-gene model for SSc by constructing ROC curves. Calibration curves were also generated to provide a comprehensive evaluation of model performance. To further explore the potential involvement of NOX4 and NEK6 in the pathogenesis of SSc-associated sarcopenia, we conducted comparative analyses across three distinct cohorts: 12 SSc patients with sarcopenia, 20 SSc patients without sarcopenia, and 20 healthy controls. This approach allowed for systematic evaluation of both diagnostic efficacy and mechanistic associations. Patients classified as having sarcopenia also met the diagnostic criteria established by the Asian Working Group for Sarcopenia 2014 (AWGS2014) (25).

### Immune infiltration landscape in SSc

To investigate immune infiltration patterns in the GSE181549 dataset, we utilized the CIBERSORT algorithm to perform deconvolution analysis, quantifying the relative proportions of 22 immune cell subpopulations (26). The distribution of these immune subsets was visualized using a stacked bar chart generated with the “ggplot2 (v3.3.0)” package. Comparative analysis between SSc patients and healthy controls was then performed. Spearman’s rank correlation coefficients were computed to evaluate associations between candidate diagnostic genes and the abundance of immune cells. We also have applied the Benjamini-Hochberg procedure to adjust the p-values for controlling the FDR across the multiple comparisons. Hierarchical clustering heatmaps were constructed using the “pheatmap” package to visualize the relationships between the two conserved genes and immune cell composition. The adjusted P-values < 0.05 were considered statistically significant.

### Statistical analysis

All statistical analyses and visualizations were performed using R software (version 4.2.1). Differences in gene expression levels and immune cell fractions between clinical groups were assessed using a two-sided Wilcoxon test. Correlation analyses were conducted using Spearman’s rank test. A P-value < 0.05 was considered indicative of statistical significance.

## Results

### Identification of DEGs and intersecting genes in SSc and sarcopenia

**Figure 2:**
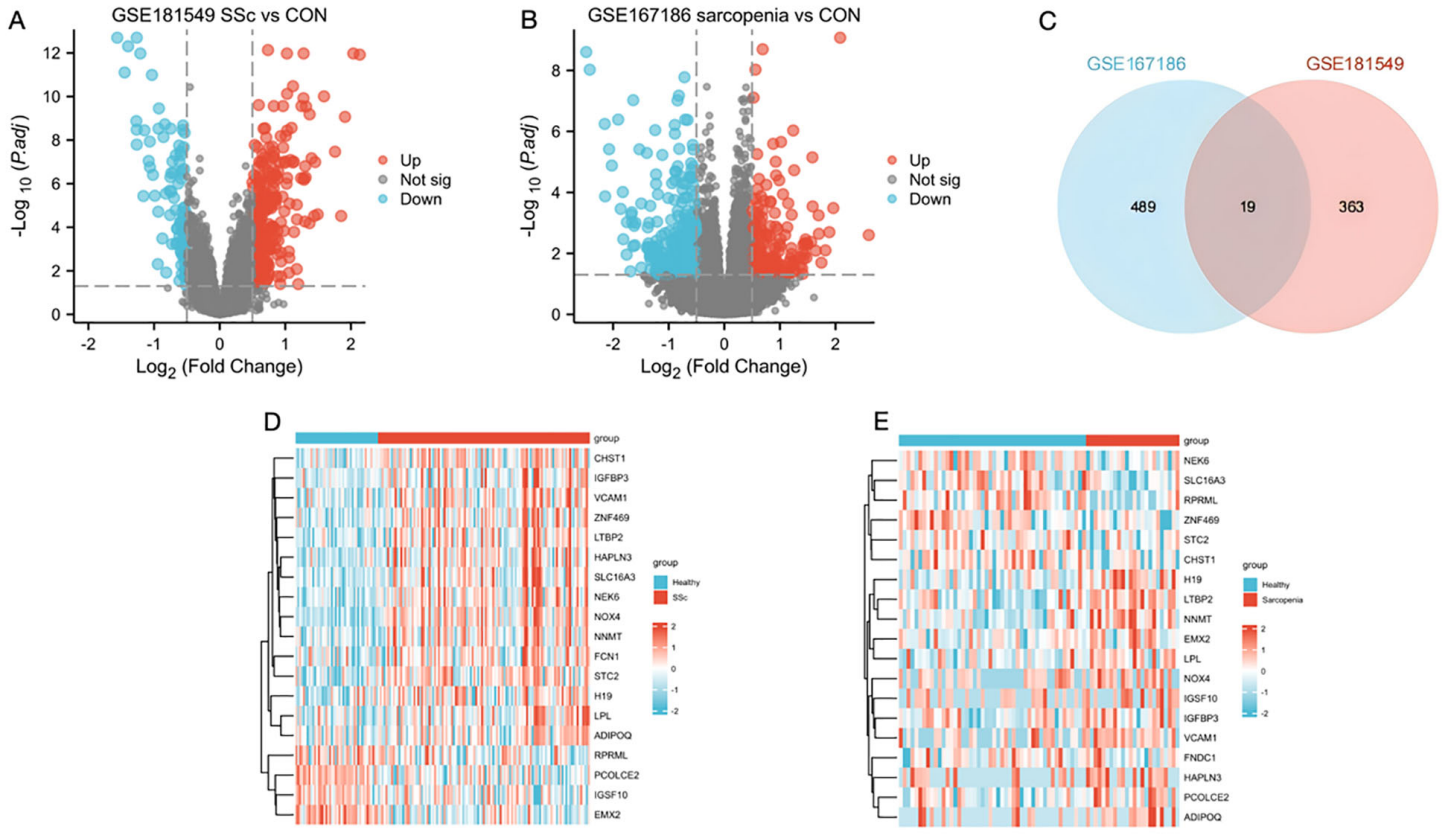
Identification of DEGs and the common genes in SSc and sarcopenia. **(A)** Volcano plots showed differentially expressed genes (DEGs) of systemic sclerosis. **(B)** The volcano plot of DEGs in sarcopenia patients. **(C)** The shared DEGs between SSc and sarcopenia by overlapping the DEGs of them. **(D)** The shared DEG heatmap in SSc group. **(E)** The shared DEG heatmap in sarcopenia group. CON, Control; SSc, systemic sclerosis.

Analysis of the SSc dataset GSE181549 revealed 382 DEGs in the SSc group (**Figure 2A**). To identify DEGs associated with sarcopenia, skeletal muscle samples from 24 sarcopenia patients and 48 healthy controls were analyzed using dataset GSE167186, yielding 508 DEGs (**Figure 2B**). Intersecting the DEGs from both datasets identified 19 shared genes (**Figure 2C**), providing valuable insights into the common molecular mechanisms underlying SSc and sarcopenia. Heatmaps illustrating these 19 intersecting genes in both datasets (**Figures 2D, E**) demonstrated clear differences in gene expression patterns between disease and control groups. The 19 shared genes were: FCN1, NOX4, STC2, NEK6, LTBP2, H19, HAPLN3, SLC16A3, IGFBP3, IGSF10, VCAM1, PCOLCE2, NNMT, EMX2, CHST1, LPL, RPRML, ADIPOQ, ZNF469.

### Identification of target genes through machine learning

**Figure 3:**
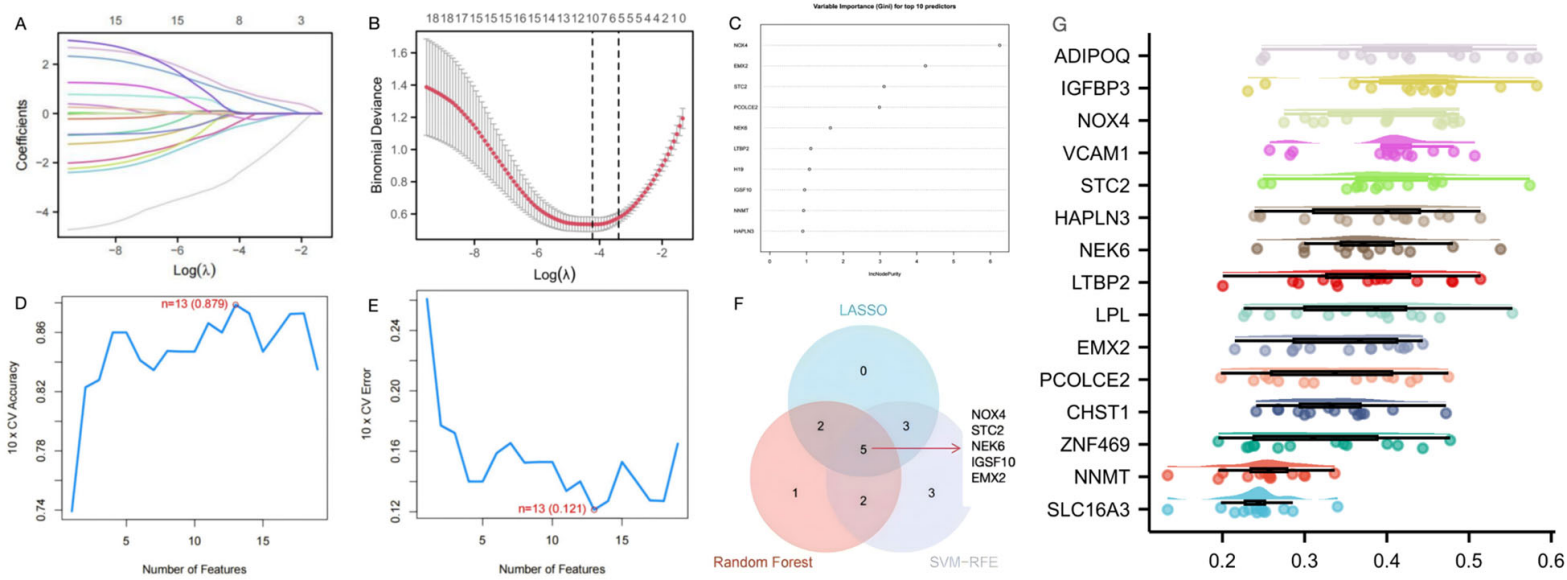
Screening of diagnostic genes by machine learning **(A)** Path diagram of the LASSO regression model. **(B)** The optimal tuning parameter selection map of LASSO algorithm. **(C)** SSc top-10 genes according to their discriminant ability in the RF algorithm. **(D)** SVM-RFE 10-fold cross-validation accuracy. **(E)** SVM-RFE 10-fold cross-validation error rate. **(F)** The Venn diagram showed five candidate diagnostic genes in SSc by intersecting the results of there algorithms. **(G)** Cloud and rain map of friends analysis of 19 common DEGs in GSE181549 and GSE167186.

To further identify candidate diagnostic biomarkers capable of discriminating between disease and control states, three machine learning algorithms were applied to the 19 shared genes: LASSO regression, RF, and SVM-RFE. In the SSc group, LASSO coefficient profiling (**Figure 3A**) and optimal lambda selection (**Figure 3B**) indicated that the tuning parameter λ was 0.0146, which balanced model complexity and predictive performance. The LASSO model identified 10 candidate genes. These 19 genes were also evaluated using the RF classifier, and the top 10 genes were ranked by importance (**Figure 3C**). To further improve diagnostic accuracy, the SVM-RFE method was employed. Model accuracy peaked at 0.879 with 13 genes (**Figure 3D**), and the minimum error rate was 0.121 (**Figure 3E**). Ultimately, five genes—NOX4, STC2, NEK6, IGSF10, and EMX2—were identified through the intersection of all three algorithms (**Figure 3F**). Functional prioritization by the “Friends” analysis confirmed that NOX4, STC2, NEK6, and EMX2 were among the top 10 functionally relevant genes (**Figure 3G**).

### Screening of diagnostic biomarkers and construction of a predictive model in SSc and sarcopenia datasets

**Figure 4:**
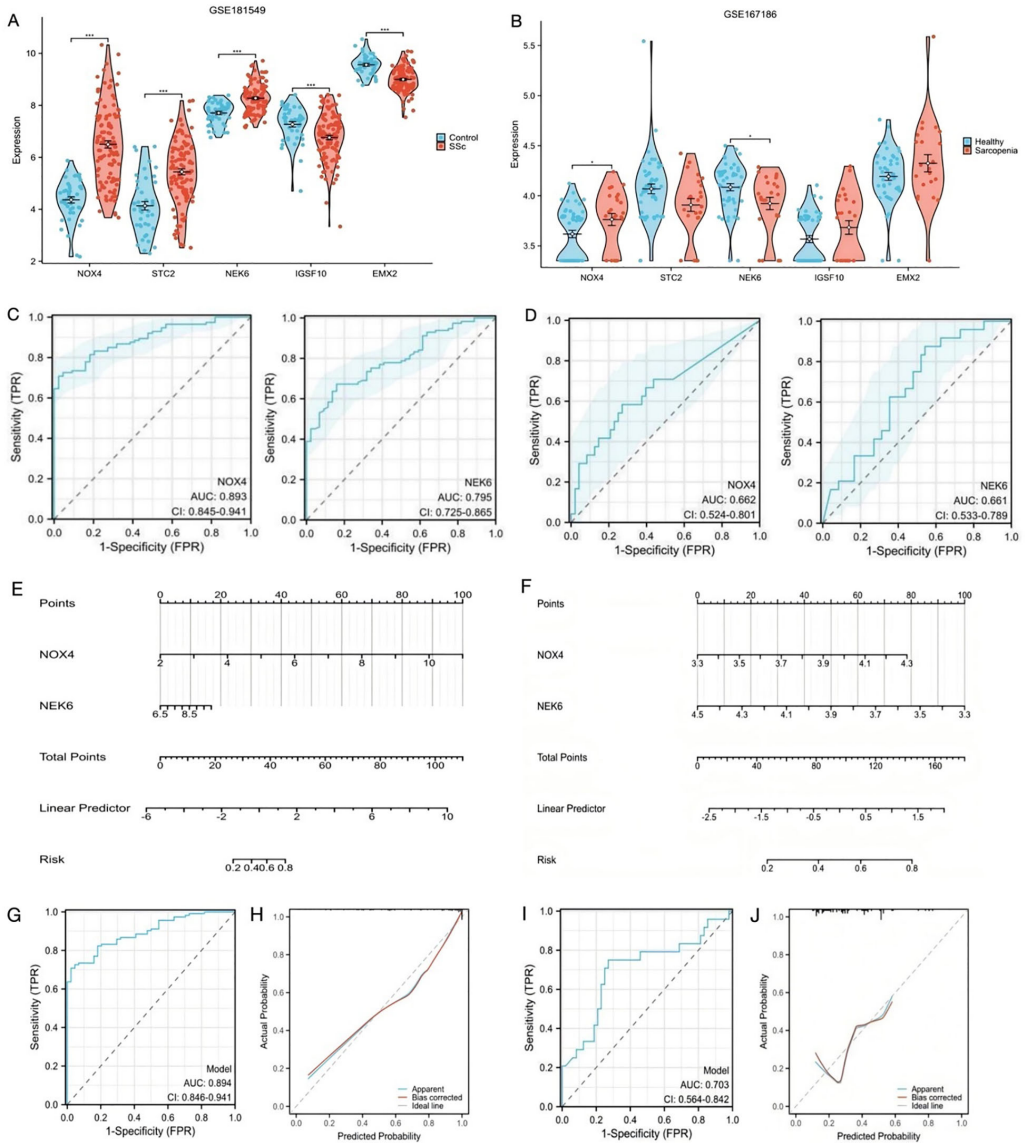
Construction of predictive scoring model **(A)** The expression level of the shared genes in GSE181549 for SSc. **(B)** The expression level of the shared genes in GSE167186 for sarcopenia. **(C)** ROC curves of NOX4 and NEK6 for SSc. **(D)** ROC curves of NOX4 and NEK6 for sarcopenia. **(E, F)** Nomogram predicting the probability of SSc and sarcopenia. **(G, H)** ROC curve and Calibration curves of risk model in GSE181549. **(I, J)** ROC curve and Calibration curves of risk model in GSE167186.

For the five candidate biomarkers, single-gene expression analyses were conducted. In the GSE181549 dataset, the expression levels of NOX4, STC2, and NEK6 were significantly upregulated in the SSc group relative to controls, whereas IGSF10 and EMX2 were downregulated (**Figure 4A**). In the sarcopenia dataset GSE167186, NOX4 expression was elevated, and NEK6 expression was reduced in sarcopenia patients compared with controls (**Figure 4B**). The expression of STC2, IGSF10, and EMX2 showed no significant differences.

The objective of this study was to identify biomarkers with relevance to both SSc and sarcopenia. Since NOX4 and NEK6 were differentially expressed in both datasets, they were selected for further validation. ROC curve analysis was used to evaluate their diagnostic performance. In the SSc dataset, the AUC was 0.893 (95% CI: 0.845 – 0.941) for NOX4 and 0.795 (95% CI: 0.725 – 0.865) for NEK6 (**Figure 4C**), indicating robust diagnostic potential. In the sarcopenia dataset, the AUC for NOX4 was 0.662 (95% CI: 0.524 – 0.801), and for NEK6, it was 0.661 (95% CI: 0.533 – 0.789) (**Figure 4D**), suggesting limited individual predictive efficacy in sarcopenia.

To identify more effective diagnostic indicators, this study first confirmed that NOX4 and NEK6 serve as independent risk factors for both SSc and sarcopenia through multivariate regression analysis. A risk score model was subsequently constructed by weighting the normalized expression levels of NOX4 and NEK6 using the regression coefficients derived from multivariate logistic regression analysis. As shown in **Figure 4E**, the SSc risk score was calculated as: normalized expression of NOX4 × 1.624 + normalized expression of NEK6 × 0.704. For sarcopenia (**Figure 4F**), the risk score was: normalized expression of NOX4 × 2.229 + normalized expression of NEK6 × (–2.369). The ROC curves indicated high predictive accuracy for both diseases (**Figures 4G, I**). The C-index of the two-gene risk model exceeded that of the single-gene models, indicating superior predictive performance. The bias-corrected calibration curves were closely aligned with the ideal curve (**Figures 4H, J**). Additionally, to mitigate the risk of overfitting, we conducted internal cross-validation within the GSE181549 dataset. The results, as shown in **Supplementary Table 1**, demonstrated an AUC of 0.890, which further confirms the model’s robust classification performance and stability.

### Validation of biomarkers and evaluation of predictive model performance in our cohort

**Figure 5:**
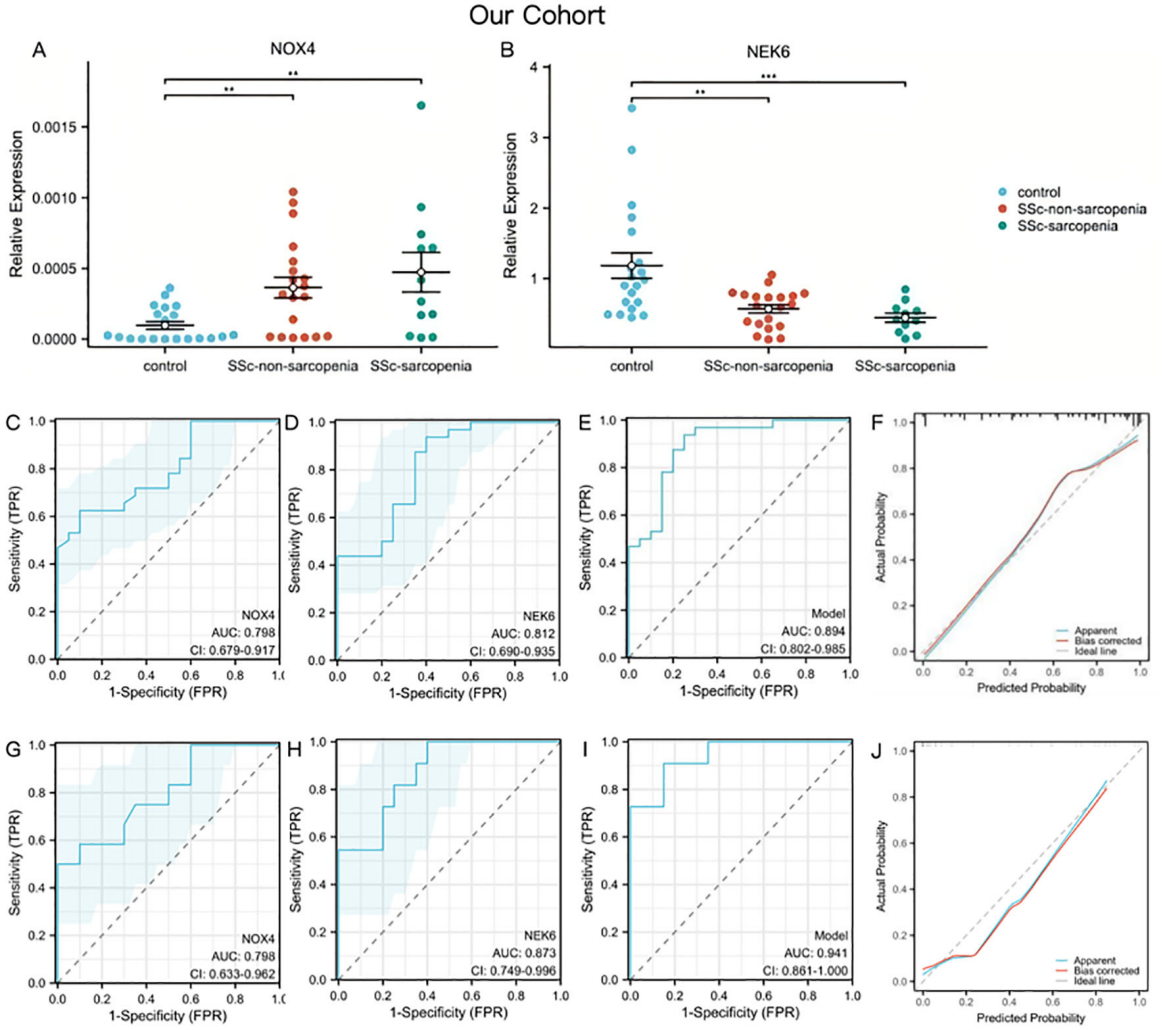
Genes validation and assessment of diagnostic effectiveness of predictive model in SSc-sarcopenia **(A, B)** The expression of NOX4 and NEK6 in SSc-sarcopenia by PCR in our cohort. **(C–E)** ROC curves of NOX4, NEK6 and the two-gene risk model for SSc in our cohort. **(F)** Calibration curves of the SSc risk model. **(G–I)** ROC curves of NOX4, NEK6 and the two-gene risk model for SSc-sarcopenia in our cohort. **(J)** Calibration curves of the SSc-sarcopenia risk model. Statistical significance **P <0.01, ***P <0.001.

To further validate the expression levels of NOX4 and NEK6, whole blood samples were collected from 20 healthy controls and 32 SSc patients, including 12 with sarcopenia and 20 without. The results revealed that NOX4 expression was elevated in both the SSc sarcopenia and non-sarcopenia groups compared to healthy controls (**Figure 5A**). In contrast, NEK6 expression was reduced in both patient groups (**Figure 5B**). However, no statistically significant differences in NOX4 and NEK6 expression were observed between the SSc sarcopenia and non-sarcopenia groups.

Using PCR data from our cohort as the validation set, **Figures 5C, D** demonstrated satisfactory diagnostic performance of NOX4 (AUC = 0.798, 95% CI: 0.679 – 0.917) and NEK6 (AUC = 0.812, 95% CI: 0.690 – 0.935) for SSc. The two-gene risk score showed superior predictive capability, achieving an AUC of 0.894 (**Figure 5E**). Moreover, the bias-corrected calibration plot closely approximated the ideal curve (**Figure 5F**). To further assess the roles of NOX4 and NEK6 in the pathogenesis of SSc-associated sarcopenia, ROC curves were generated to evaluate their diagnostic performance. NOX4 (AUC = 0.798, 95% CI: 0.633 – 0.962) and NEK6 (AUC = 0.873, 95% CI: 0.749 – 0.996) exhibited high diagnostic accuracy for SSc-associated sarcopenia (**Figures 5G, H**). The two-gene model showed the highest discriminative performance, with an AUC of 0.941 (95% CI: 0.861 – 1.000), supporting its favorable predictive efficacy (**Figure 5I**). The calibration plot demonstrated excellent agreement between predicted probabilities and observed outcomes after bias correction, closely aligning with the ideal curve across the full probability range (**Figure 5J**).

To assess the diagnostic utility of NOX4 and NEK for sarcopenia in SSc, we performed a Pearson correlation analysis with ASMI. ASMI and NOX4 have a substantial negative connection (r=-0.31, p=0.041). Additionally, there is a positive connection (r = 0.45, p = 0.01) between ASMI and NEK6.

### Immune infiltration landscape in SSc

**Figure 6:**
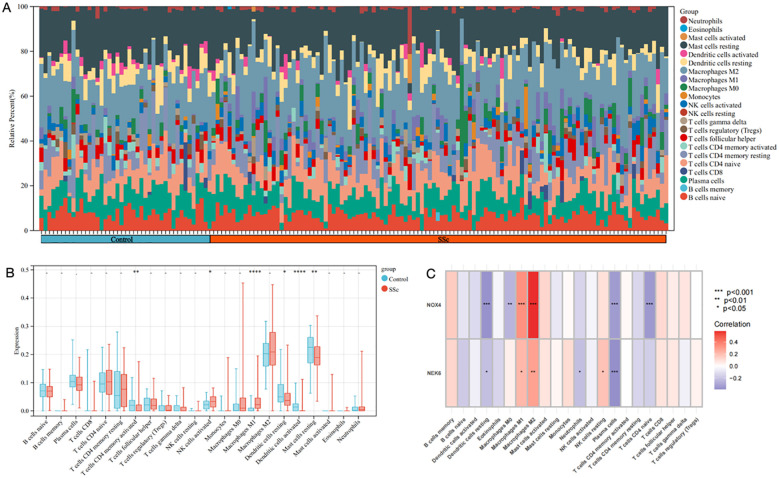
The immune infiltration landscape of SSc. **(A)** The heatmap showing the distribution of different immune cell types in skin samples of SSc and controls. **(B)** Infiltrating difference of immune cells between SSc patients and controls in the box plot. **(C)** The heatmaps showed the correlation of two DEGs with immune cell infiltration in SSc patients. Statistical significance. *P <0.05, **P <0.01, ***P <0.001 and **** P<0.0001.

A comprehensive analysis was conducted to characterize the immune cell infiltration patterns and their functional relevance in SSc and control groups. **Figure 6A** illustrates the relative proportions of immune cell subsets, indicating significant alterations in the SSc group. The CIBERSORT algorithm was employed to calculate the proportions of 22 immune cell types in each sample from the GSE181549 dataset. Notably, several immune cells, including activated CD4^+^ memory T cells, activated NK cells, M1 macrophages, and both resting and activated dendritic cells and mast cells, exhibited significantly increased infiltration in the SSc group compared to controls (**Figure 6B**).

**Figure 7:**
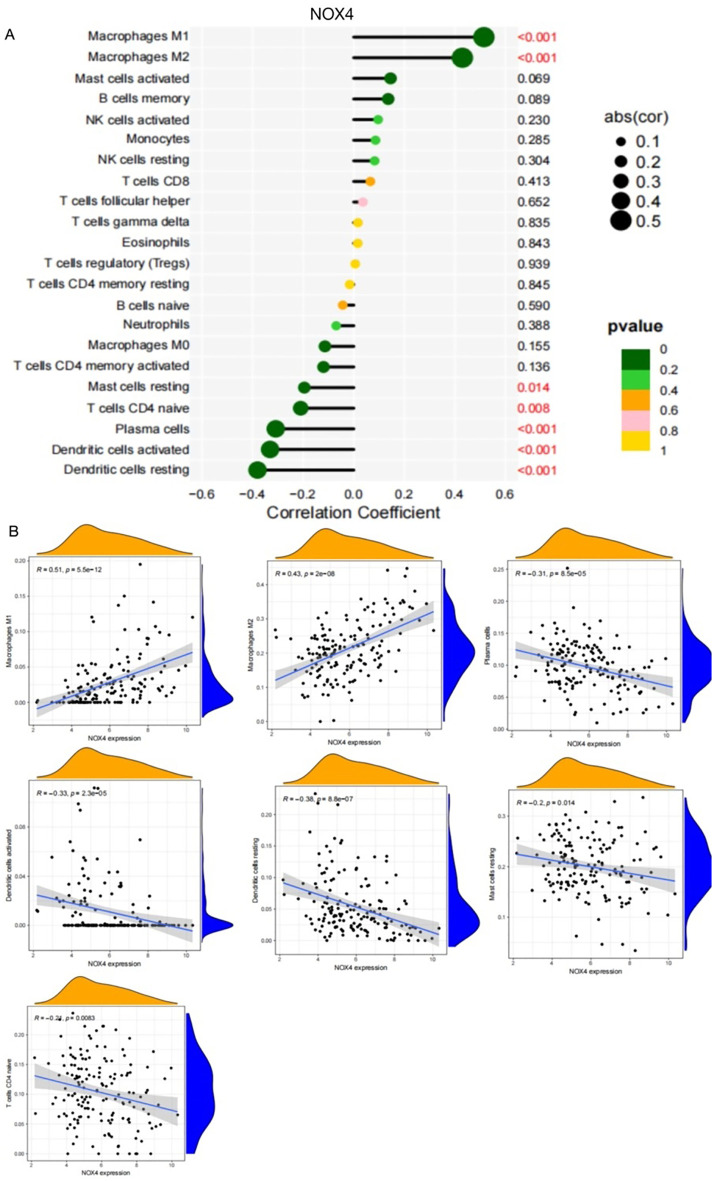
Spearman correlation between NOX4 and immune cells in SSc. **(A)** Correlation Coefficient of NOX4 and immune cells **(B)** Representative correlations between NOX4 and selected immune cell subtypes.

**Figure 8:**
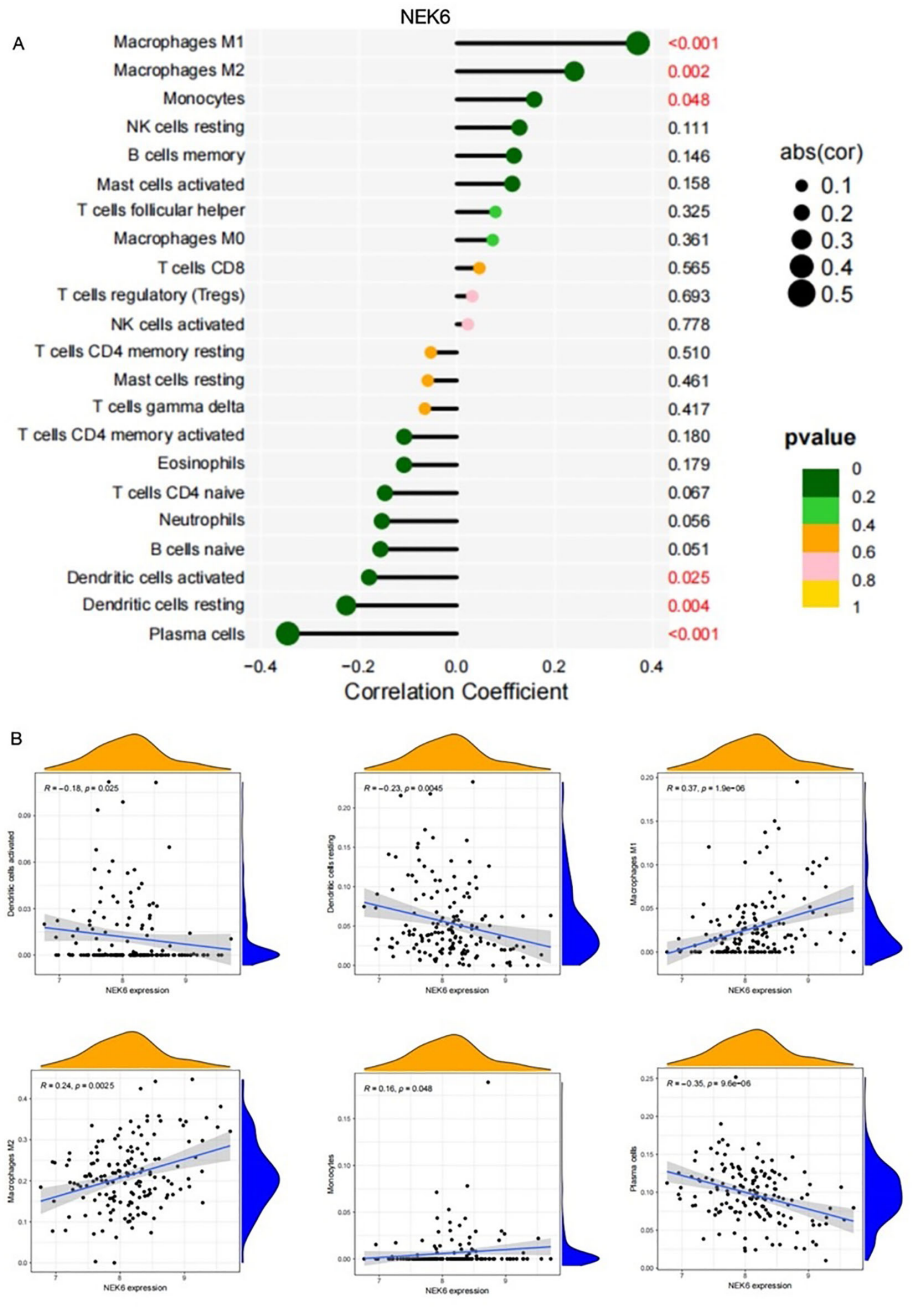
Correlation between NEK6 and immune cells in SSc.**(A)** Correlation Coefficient of NEK6 and immune cells **(B)** Representative correlations between NEK6 and selected immune cell subtypes.

As shown in **Figure 6C**, NOX4 and NEK6 were significantly correlated with infiltration levels of various immune cells in the SSc cohort, including plasma cells, dendritic cells, naive CD4^+^ T cells, macrophages, NK cells, and neutrophils. Specifically, NOX4 displayed strong positive correlations with both M1 and M2 macrophages, and significant negative correlations with resting dendritic cells, activated dendritic cells, plasma cells, activated CD4^+^ memory T cells, and resting mast cells (**Figure 7A**). Correlation coefficients greater than 0.3 were observed between NOX4 and M1/M2 macrophages, plasma cells, and both resting and activated dendritic cells (**Figure 7B**). Similarly, NEK6 showed positive correlations with macrophages and monocytes, and negative correlations with plasma cells, resting and activated dendritic cells, and mast cells (**Figure 8A**). Significant correlations (R > 0.3) were noted between NEK6 and M1 macrophages as well as plasma cells (**Figure 8B**). These findings suggest that NOX4 and NEK6 may influence the pathophysiological progression of SSc by modulating immune cell infiltration, thereby offering novel perspectives for immunomodulatory therapeutic strategies.

## Discussion

This study identified NOX4 and NEK6 as dual diagnostic biomarkers for SSc-associated sarcopenia through integrative bioinformatics and machine learning approaches. The dual-gene risk score model exhibited strong diagnostic efficacy, with AUC values exceeding 0.89 in both discovery and validation cohorts. These findings provide valuable insights into the shared molecular mechanisms underlying SSc and sarcopenia and propose a clinically applicable tool for early disease detection.

The model positions NOX4 and NEK6 as pivotal contributors to SSc-associated sarcopenia. NOX4, an isoform of NADPH oxidase, is known to regulate intracellular reactive oxygen species (ROS) levels (27). While ROS play critical roles in cellular signaling, excessive ROS induce oxidative stress, resulting in cellular damage and dysfunction (28). Oxidative stress is considered a central pathogenic factor in SSc, promoting inflammation, immune dysregulation, and fibrosis. Numerous studies have demonstrated that ROS overproduction is closely associated with these pathological processes in SSc (29). Our previous study confirmed elevated expression of IL - 6 and NOX4 in peripheral blood mononuclear cells (PBMCs) from SSc patients using RT-PCR (30). Additionally, a recent study found that NOX4 was upregulated in the skin tissues of SSc patients, with its expression consistently validated across four independent datasets (31).

Elevated NOX4 expression may contribute to muscle atrophy via ROS-mediated mitochondrial dysfunction and protein degradation, both of which are implicated in the pathogenesis of sarcopenia (32). Hammers et al. (33) reported increased NOX4 levels in the muscle tissue of myotonic dystrophy mouse models and Duchenne muscular dystrophy (DMD) patients. Notably, NOX4 upregulation was primarily localized to the interstitial spaces between muscle fibers. Targeting NOX4 genetically or pharmacologically significantly reduced fibrosis in atrophic respiratory and limb muscles, further supporting its role in muscle degeneration.

Conversely, NEK6, a serine/threonine kinase involved in cell cycle regulation, exhibited reduced expression in patients with SSc. Its downregulation may lead to increased reactive oxygen species (ROS) levels and DNA damage (34), thereby contributing to progressive muscle degradation. In this study, decreased NEK6 expression in the peripheral blood of SSc patients may have elevated ROS levels, subsequently promoting the pathogenesis of SSc. Several studies have confirmed that increased ROS levels mediate excessive oxidative stress, which plays a pivotal role in SSc pathogenesis (29, 35–37), consistent with our findings. Moreover, NEK6 expression was also found to be downregulated in patients with myasthenia gravis, suggesting that decreased NEK6 expression may impair muscle cell regeneration and contribute to the development of sarcopenia.

Notably, analysis of the SSc dataset (GSE181549) revealed significantly elevated NEK6 expression in lesional skin biopsies of patients with systemic sclerosis compared to healthy controls. In contrast, both sarcopenia-related datasets and our independent qPCR validation cohort demonstrated markedly reduced NEK6 expression in peripheral blood samples of SSc patients relative to healthy individuals. This discrepancy in expression patterns may be attributed primarily to tissue-specific biological differences, as the GEO dataset utilized cutaneous samples, while our validation employed peripheral blood specimens. The use of peripheral blood for biomarker validation aligns with our objective to develop minimally invasive diagnostic tools, given the clinical impracticality of repeated skin biopsies. Future investigations incorporating multi-compartmental sampling (e.g., cutaneous and circulating tissues) are warranted to elucidate the tissue-specific regulatory mechanisms governing NEK6 expression in SSc pathogenesis.

When using PCR to confirm mRNA expression, we saw a definite tendency of difference between the two patient groups, even though there were no statistically significant variations in NOX4 and NEK6 expression levels. This pattern may indicate that NOX4 and NEK6 may have an potential association between SSc and sarcopenia. However, this possible relationship has not been statistically demonstrated because of the limited sample size. In order to more precisely evaluate the variations in their expression levels and determine whether there is statistical significance, we therefore intend to increase the sample size in subsequent research on NOX4 and NEK6 in SSc associated sarcopenia.

In the sarcopenia dataset (GSE167186), the individual AUC values for NOX4 and NEK6 were 0.662 (95% CI: 0.524 – 0.801) and 0.661 (95% CI: 0.533 – 0.789) respectively. These values indicate a limited capacity for either gene to predict sarcopenia when considered in isolation. The collective AUC for the dual-gene model increased to 0.703, suggesting a marginal improvement in predictive performance when both genes are considered together. We acknowledge that these AUC values are modest and may not convey a strong predictive power. This observation could be due to several factors. First, the genetic heterogeneity and complex pathophysiology of sarcopenia may not be fully captured by only two biomarkers. Second, the relatively small sample size of the GSE167186 cohort might limit the statistical power to detect a stronger association. Third, the expression levels of NOX4 and NEK6 might be influenced by various confounding factors, such as age, sex, and comorbidities, which were not accounted for in this analysis. Despite the limitations, our findings provide a preliminary insight into the potential of NOX4 and NEK6 as biomarkers. We propose that future research, including larger sample sizes and the integration of additional biomarkers or clinical variables, could enhance the model’s predictive performance.

Systematic immune profiling revealed significant alterations in the immune microenvironment of SSc. Importantly, both NOX4 and NEK6 exhibited extensive correlations with immune cell dynamics (**Figures 6**-**8**), suggesting their potential roles as immunomodulators in SSc pathogenesis. Specifically, NOX4 demonstrated strong positive correlations with pro-inflammatory M1 and M2 macrophages (**Figures 7A, B**), implicating its involvement in macrophage polarization and fibrotic processes, which are known pathological hallmarks of SSc (38). Similarly, NEK6 showed a positive correlation with M1 macrophages and a negative association with plasma cells (**Figures 8A, B**). Previous studies have indicated that cytokines such as IL - 6 and TGF-β, produced by B cells, can induce myofibroblast differentiation. Additionally, autoantibodies can form immune complexes that bind to fibroblasts and stimulate profibrotic effects. Some autoantibody specificities, such as those targeting PDGF, observed in subsets of SSc patients, may directly bind to fibroblasts and activate them (39). Collectively, these findings suggest that NOX4 and NEK6 may contribute to SSc progression by modulating immune cell populations.

This study employed advanced computational approaches to address the heterogeneity in sarcopenia diagnosis. By integrating bioinformatics analyses with three machine learning algorithms (LASSO, RF, and SVM-RFE), we minimized selection bias and enhanced the reliability of biomarker identification. Furthermore, qPCR validation in a clinical cohort reinforced the translational relevance of our findings. Notably, the dual-gene risk score outperformed single-gene models, underscoring the value of combinatorial biomarkers in complex, multifactorial diseases. The inclusion of immune cell profiling provided additional mechanistic insight by linking gene expression markers to immune-mediated pathological processes.

Despite the robust methodology and significant findings of our study, it is crucial to address its limitations, particularly regarding sample size. A *post-hoc* power analysis based on the logistic regression model developed with the systemic sclerosis dataset GSE181549 has indicated that our current sample size of 157 is indeed less than the required 345 to achieve the desired C-index of 0.894 with adequate power. This discrepancy underscores a limitation in our study, potentially affecting the statistical power and the generalizability of our results. We propose that future studies should employ larger sample sizes to further validate our biomarkers and enhance the reliability of our predictive model. Secondly, while NOX4 and NEK6 expression is strongly linked to disease phenotypes, we couldn’t address all possible confounders. Thus, we intend to perform cellular and animal functional studies to determine these genes’ causal roles in disease development. Additionally, multi-omics approaches may be required to improve diagnostic specificity. Future studies should prioritize longitudinal cohort designs to assess the prognostic utility of NOX4 and NEK6 and to explore therapeutic strategies targeting these pathways.

## Conclusion

**Figure 9:**
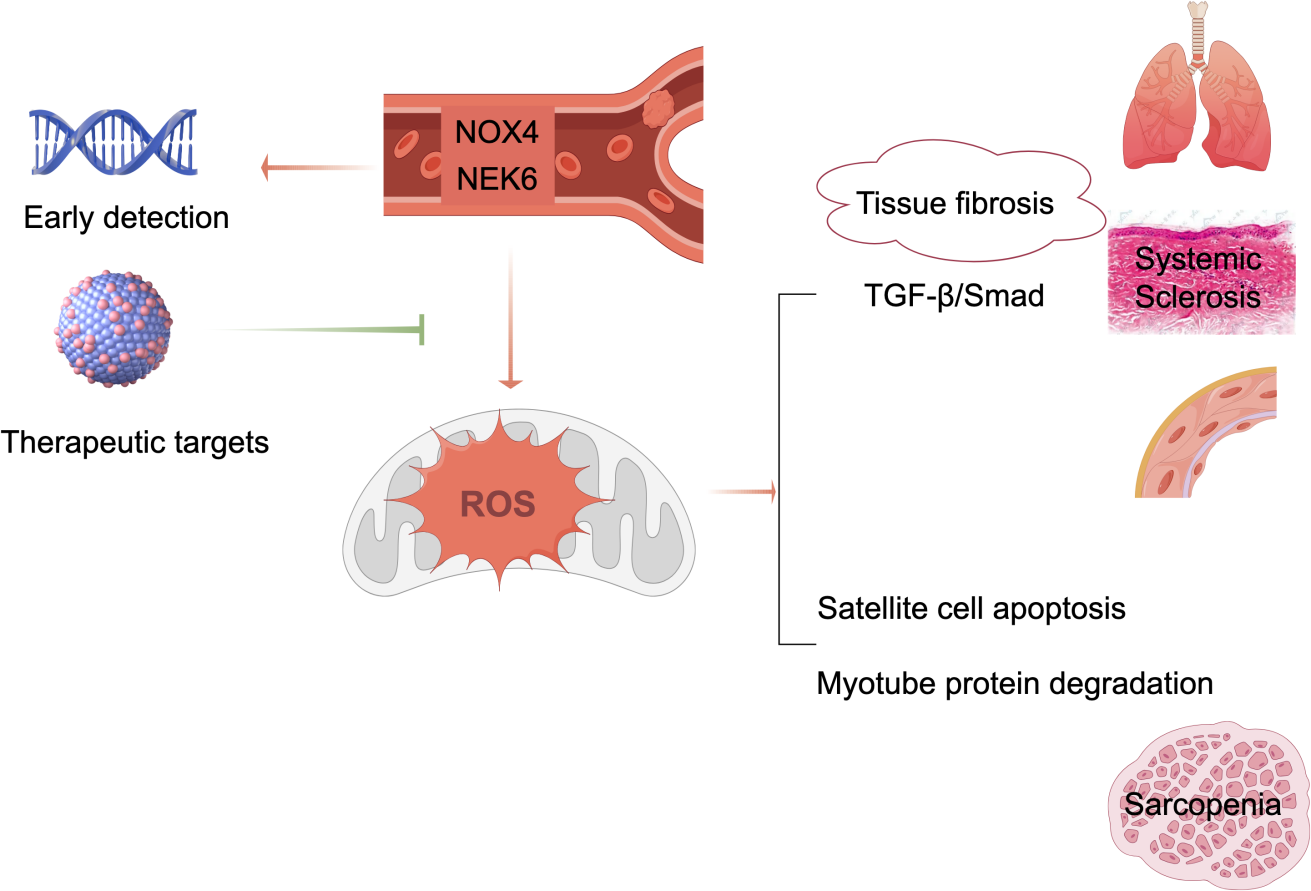
Schematic diagram of the mechanisms and proposed application.

This study pioneers the application of machine learning and bioinformatics to identify genes shared between SSc and sarcopenia. Our results establish NOX4 and NEK6 as novel diagnostic biomarkers for SSc-associated sarcopenia and propose a non-invasive strategy for its early detection. Shown in **Figure 9**, these molecules represent promising targets for future diagnostic and therapeutic applications. Validation in large-scale studies is necessary to evaluate their utility for early screening and risk stratification in SSc populations.

## Data Availability

The datasets presented in this study can be found in online repositories. The names of the repository/repositories and accession number(s) can be found in the article/**Supplementary Material**.

## References

[1] Cruz-Jentoft AJ Sayer AA. Sarcopenia. Lancet (London England). (2019) 393:2636–46. doi: 10.1016/S0140-6736(19)31138-9, PMID: 31171417

[2] Coletta G Phillips SM. An elusive consensus definition of sarcopenia impedes research and clinical treatment: A narrative review. Ageing Res Rev. (2023) 86:101883. doi: 10.1016/j.arr.2023.101883, PMID: 36792012

[3] Yuan S Larsson SC. Epidemiology of sarcopenia: Prevalence, risk factors, and consequences. Metabolism: Clin Exp. (2023) 144:155533. doi: 10.1016/j.metabol.2023.155533, PMID: 36907247

[4] Butt S Emmanuel A. Systemic sclerosis and the gut. Expert Rev Gastroenterol Hepatol. (2013) 7:331–9. doi: 10.1586/egh.13.22, PMID: 23639091

[5] Caimmi C Caramaschi P Venturini A Bertoldo E Vantaggiato E Viapiana O. Malnutrition and sarcopenia in a large cohort of patients with systemic sclerosis. Clin Rheumatol. (2018) 37:987–97. doi: 10.1007/s10067-017-3932-y, PMID: 29196890

[6] Siegert E March C Otten L Makowka A Preis E Buttgereit F. Prevalence of sarcopenia in systemic sclerosis: assessing body composition and functional disability in patients with systemic sclerosis. Nutr (Burbank Los Angeles County Calif.). (2018) 55-56:51–5. doi: 10.1016/j.nut.2018.03.046, PMID: 29960157

[7] Corallo C Fioravanti A Tenti S Pecetti G Nuti R Giordano N. Sarcopenia in systemic sclerosis: the impact of nutritional, clinical, and laboratory features. Rheumatol Int. (2019) 39:1767–75. doi: 10.1007/s00296-019-04401-w, PMID: 31372720

[8] Zuo X Li X Tang K Zhao R Wu M Wang Y. Sarcopenia and cardiovascular diseases: A systematic review and meta-analysis. J cachexia sarcopenia Muscle. (2023) 14:1183–98. doi: 10.1002/jcsm.13221, PMID: 37002802 PMC10235887

[9] Hongkanjanapong S Pongkulkiat P Mahakkanukrauh A Suwannaroj S Foocharoen C. Clinical outcomes and associated factors with mortality in systemic sclerosis patients with sarcopenia. Am J Med Sci. (2025) 369:35–43. doi: 10.1016/j.amjms.2024.07.025, PMID: 39033816

[10] Sabatino A D’Alessandro C Regolisti G di Mario F Guglielmi G Bazzocchi A. Muscle mass assessment in renal disease: the role of imaging techniques. Quantitative Imaging Med Surg. (2020) 10:1672–86. doi: 10.21037/qims.2020.03.05, PMID: 32742960 PMC7378093

[11] Castillo Castro C González Arellanes R Camacho Mondragón CG Farfán Esponda HR Del Razo Olvera FM Aguilar Salinas CA. Agreement between bioelectrical impedance analysis and dual-energy X-ray absorptiometry to estimate fat mass in hispanic adults with type 2 diabetes mellitus: A cross-sectional study. Clin Med Insights Endocrinol Diabetes. (2024) 17:11795514241274691. doi: 10.1177/11795514241274691, PMID: 39224772 PMC11367586

[12] Coelho-Júnior HJ Calvani R Picca A Marzetti E. Are sit-to-stand and isometric handgrip tests comparable assessment tools to identify dynapenia in sarcopenic people? Arch gerontology geriatrics. (2023) 114:105059. doi: 10.1016/j.archger.2023.105059, PMID: 37295058

[13] Warden SJ Liu Z Moe SM. Sex- and age-specific centile curves and downloadable calculator for clinical muscle strength tests to identify probable sarcopenia. Phys Ther. (2022) 102:pzab299. doi: 10.1093/ptj/pzab299, PMID: 34972866 PMC9005054

[14] Wiedmer P Jung T Castro JP Pomatto LCD Sun PY Davies KJA. Sarcopenia - Molecular mechanisms and open questions. Ageing Res Rev. (2021) 65:101200. doi: 10.1016/j.arr.2020.101200, PMID: 33130247

[15] Liu G Jiang S Xie W Liu X Yang G Lu W. Biomarkers for sarcopenia, muscle mass, muscle strength, and physical performance: an umbrella review. J Trans Med. (2025) 23:650. doi: 10.1186/s12967-025-06575-3, PMID: 40506715 PMC12160432

[16] Sakuma K Hamada K Yamaguchi A Aoi W. Current nutritional and pharmacological approaches for attenuating sarcopenia. Cells. (2023) 12:2422. doi: 10.3390/cells12192422, PMID: 37830636 PMC10572610

[17] Nasso R D’Errico A Motti ML Masullo M Arcone R. Dietary protein and physical exercise for the treatment of sarcopenia. Clinics Pract. (2024) 14:1451–67. doi: 10.3390/clinpract14040117, PMID: 39194921 PMC11352344

[18] Hu J Wang Y Ji X Zhang Y Li K Huang F. Non-pharmacological strategies for managing sarcopenia in chronic diseases. Clin Interventions Aging. (2024) 19:827–41. doi: 10.2147/CIA.S455736, PMID: 38765795 PMC11102744

[19] Barrett T Troup DB Wilhite SE Ledoux P Rudnev D Evangelista C. NCBI GEO: mining tens of millions of expression profiles–database and tools update. Nucleic Acids Res. (2007) 35:D760–5. doi: 10.1093/nar/gkl887, PMID: 17099226 PMC1669752

[20] Skaug B Lyons MA Swindell WR Salazar GA Wu M Tran TM. Large-scale analysis of longitudinal skin gene expression in systemic sclerosis reveals relationships of immune cell and fibroblast activity with skin thickness and a trend towards normalisation over time. Ann Rheum Dis. (2022) 81:516–23. doi: 10.1136/annrheumdis-2021-221352, PMID: 34937693 PMC8956967

[21] Perez K Ciotlos S McGirr J Limbad C Doi R Nederveen JP. Single nuclei profiling identifies cell specific markers of skeletal muscle aging, frailty, and senescence. Aging. (2022) 14:9393–422. doi: 10.18632/aging.204435, PMID: 36516485 PMC9792217

[22] Preliminary criteria for the classification of systemic sclerosis (scleroderma). Subcommittee for scleroderma criteria of the American Rheumatism Association Diagnostic and Therapeutic Criteria Committee. Arthritis Rheum. (1980) 23:581–90. doi: 10.1002/art.1780230510, PMID: 7378088

[23] Cruz-Jentoft AJ Baeyens JP Bauer JM Boirie Y Cederholm T Landi F. Sarcopenia: European consensus on definition and diagnosis: Report of the European Working Group on Sarcopenia in Older People. Age Ageing. (2010) 39:412–23. doi: 10.1093/ageing/afq034, PMID: 20392703 PMC2886201

[24] Huang ML Hung YH Lee WM Li RK Jiang BR. SVM-RFE based feature selection and Taguchi parameters optimization for multiclass SVM classifier. ScientificWorldJournal. (2014) 2014:795624. doi: 10.1155/2014/795624, PMID: 25295306 PMC4175386

[25] Chen LK Liu LK Woo J Assantachai P Auyeung TW Bahyah KS. Sarcopenia in Asia: consensus report of the Asian Working Group for Sarcopenia. J Am Med Directors Assoc. (2014) 15:95–101. doi: 10.1016/j.jamda.2013.11.025, PMID: 24461239

[26] Newman AM Liu CL Green MR Gentles AJ Feng W Xu Y. Robust enumeration of cell subsets from tissue expression profiles. Nat Methods. (2015) 12:453–7. doi: 10.1038/nmeth.3337, PMID: 25822800 PMC4739640

[27] Wang S Nikamo P Laasonen L Gudbjornsson B Ejstrup L Iversen L. Rare coding variants in NOX4 link high ROS levels to psoriatic arthritis mutilans. EMBO Mol Med. (2024) 16:596–615. doi: 10.1038/s44321-024-00035-z, PMID: 38379095 PMC10940640

[28] Andrieux P Chevillard C Cunha-Neto E Nunes JPS. Mitochondria as a cellular hub in infection and inflammation. Int J Mol Sci. (2021) 22:11338. doi: 10.3390/ijms222111338, PMID: 34768767 PMC8583510

[29] Doridot L Jeljeli M Chêne C Batteux F. Implication of oxidative stress in the pathogenesis of systemic sclerosis via inflammation, autoimmunity and fibrosis. Redox Biol. (2019) 25:101122. doi: 10.1016/j.redox.2019.101122, PMID: 30737171 PMC6859527

[30] Wu C Liu J Chen Z Wu Y Gao F. Comprehensive analysis of ferroptosis-related hub gene signatures as a potential pathogenesis and therapeutic target for systemic sclerosis: A bioinformatics analysis. Int J immunopathology Pharmacol. (2023) 37:3946320231187783. doi: 10.1177/03946320231187783, PMID: 37403234 PMC10331081

[31] Zheng L Wu Q Chen S Wen J Dong F Meng N. Development and validation of a new diagnostic prediction model of ENHO and NOX4 for early diagnosis of systemic sclerosis. Front Immunol. (2024) 15:1273559. doi: 10.3389/fimmu.2024.1273559, PMID: 38348042 PMC10859860

[32] Ristow M Zarse K Oberbach A Klöting N Birringer M Kiehntopf M. Antioxidants prevent health-promoting effects of physical exercise in humans. Proc Natl Acad Sci United States America. (2009) 106:8665–70. doi: 10.1073/pnas.0903485106, PMID: 19433800 PMC2680430

[33] Hammers DW. NOX4 inhibition promotes the remodeling of dystrophic muscle. JCI Insight. (2022) 7:e158316. doi: 10.1172/jci.insight.158316, PMID: 36278481 PMC9714779

[34] Panchal NK Mohanty S Prince SE. NIMA-related kinase-6 (NEK6) as an executable target in cancer. Clin Trans Oncol. (2023) 25:66–77. doi: 10.1007/s12094-022-02926-4, PMID: 36074296

[35] Ames PRJ Bucci T Merashli M Amaral M Arcaro A Gentile F. Oxidative/nitrative stress in the pathogenesis of systemic sclerosis: are antioxidants beneficial? Free Radical Res. (2018) 52:1063–82. doi: 10.1080/10715762.2018.1525712, PMID: 30226391

[36] Piera-Velazquez S Jimenez SA. Role of cellular senescence and NOX4-mediated oxidative stress in systemic sclerosis pathogenesis. Curr Rheumatol Rep. (2015) 17:473. doi: 10.1007/s11926-014-0473-0, PMID: 25475596 PMC6779060

[37] Grygiel-Górniak B Puszczewicz M. Oxidative damage and antioxidative therapy in systemic sclerosis. Mediators Inflammation. (2014) 2014:389582. doi: 10.1155/2014/389582, PMID: 25313270 PMC4172878

[38] Mohamed ME Gamal RM El-Mokhtar MA Hassan AT Abozaid HSM Ghandour AM. Peripheral cells from patients with systemic sclerosis disease co-expressing M1 and M2 monocyte/macrophage surface markers: Relation to the degree of skin involvement. Hum Immunol. (2021) 82:634–9. doi: 10.1016/j.humimm.2021.03.009, PMID: 34020830

[39] Beesley CF Goldman NR Taher TE Denton CP Abraham DJ Mageed RA. Dysregulated B cell function and disease pathogenesis in systemic sclerosis. Front Immunol. (2023) 13:999008. doi: 10.3389/fimmu.2022.999008, PMID: 36726987 PMC9885156

