# Supplementary material for: Identification of crosstalk genes and diagnostic biomarkers in systemic sclerosis associated sarcopenia through integrative analysis and machine learning
Source: Front Immunol. 2025 Sep 5;16:1642806. doi: 10.3389/fimmu.2025.1642806 (PMC12446289; doi:10.3389/fimmu.2025.1642806)
Supplement: Supplementary file 1 [file Table1.docx]

Supplementary Table S1 Comparison of results by five-fold cross validation in the GSE181549.

| Item | Dxy | AUC | R^2^ | Brier |
| --- | --- | --- | --- | --- |
| Original Value | 0.788 | 0.894 | 0.561 | 0.119 |
| Cross Validation Value | 0.780 | 0.890 | 0.480 | 0.127 |

Note: Dxy：Discriminant index；AUC：Area Under the Curve；R2：Coefficient of Determination；Brier: Brier score.
